# Bridging Data Silos in Oncology with Modular Software for Federated Analysis on Fast Healthcare Interoperability Resources: Multisite Implementation Study

**DOI:** 10.2196/65681

**Published:** 2025-04-15

**Authors:** Jasmin Ziegler, Marcel Pascal Erpenbeck, Timo Fuchs, Anna Saibold, Paul-Christian Volkmer, Guenter Schmidt, Johanna Eicher, Peter Pallaoro, Renata De Souza Falguera, Fabio Aubele, Marlien Hagedorn, Ekaterina Vansovich, Johannes Raffler, Stephan Ringshandl, Alexander Kerscher, Julia Karolin Maurer, Brigitte Kühnel, Gerhard Schenkirsch, Marvin Kampf, Lorenz A Kapsner, Hadieh Ghanbarian, Helmut Spengler, Iñaki Soto-Rey, Fady Albashiti, Dirk Hellwig, Maximilian Ertl, Georg Fette, Detlef Kraska, Martin Boeker, Hans-Ulrich Prokosch, Christian Gulden

**Affiliations:** 1 Medical Center for Information and Communication Technology Universitätsklinikum Erlangen Erlangen Germany; 2 Bavarian Cancer Research Center (BZKF) Erlangen Germany; 3 Medical Informatics Friedrich-Alexander-Universität Erlangen-Nürnberg Erlangen Germany; 4 Department of Nuclear Medicine University Hospital Regensburg Regensburg Germany; 5 Medical Data Integration Center University Hospital Regensburg Regensburg Germany; 6 Department of Information Technology University Hospital Regensburg Regensburg Germany; 7 Comprehensive Cancer Center Mainfranken University Hospital Würzburg Würzburg Germany; 8 Data Integration Center University Hospital Würzburg Würzburg Germany; 9 Institute for Artificial Intelligence and Informatics in Medicine, Klinikum rechts der Isar School of Medicine and Health Technical University of Munich Munich Germany; 10 Data Integration Center, Klinikum rechts der Isar School of Medicine and Health Technical University of Munich Munich Germany; 11 Section of Precision Psychiatry Clinic for Psychiatry and Psychotherapy Ludwig-Maximilians-Universität München Munich Germany; 12 Medical Data Integration Center LMU University Hospital, Ludwig-Maximilians-Universität München Munich Germany; 13 Digital Medicine University Hospital of Augsburg Augsburg Germany; 14 Department of Medicine Data Integration Center Philipps-University Marburg Marburg Germany; 15 University Cancer Center Regensburg University Hospital Regensburg Regensburg Germany; 16 Comprehensive Cancer Center Munich, Klinikum rechts der Isar Technical University of Munich Munich Germany; 17 Comprehensive Cancer Center Augsburg University Hospital of Augsburg Augsburg Germany; 18 Institute of Radiology, Uniklinikum Erlangen Friedrich-Alexander-Universität Erlangen-Nürnberg Erlangen Germany

**Keywords:** real-world data, real-world evidence, oncology, electronic health records, federated analysis, HL7 FHIR, cancer registries, interoperability, observational research network

## Abstract

**Background:**

Real-world data (RWD) from sources like administrative claims, electronic health records, and cancer registries offer insights into patient populations beyond the tightly regulated environment of randomized controlled trials. To leverage this and to advance cancer research, 6 university hospitals in Bavaria have established a joint research IT infrastructure.

**Objective:**

This study aimed to outline the design, implementation, and deployment of a modular data transformation pipeline that transforms oncological RWD into a Health Level 7 (HL7) Fast Healthcare Interoperability Resources (FHIR) format and then into a tabular format in preparation for a federated analysis (FA) across the 6 Bavarian Cancer Research Center university hospitals.

**Methods:**

To harness RWD effectively, we designed a pipeline to convert the oncological basic dataset (oBDS) into HL7 FHIR format and prepare it for FA. The pipeline handles diverse IT infrastructures and systems while maintaining privacy by keeping data decentralized for analysis. To assess the functionality and validity of our implementation, we defined a cohort to address two specific medical research questions. We evaluated our findings by comparing the results of the FA with reports from the Bavarian Cancer Registry and the original data from local tumor documentation systems.

**Results:**

We conducted an FA of 17,885 cancer cases from 2021/2022. Breast cancer was the most common diagnosis at 3 sites, prostate cancer ranked in the top 2 at 4 sites, and malignant melanoma was notably prevalent. Gender-specific trends showed larynx and esophagus cancers were more common in males, while breast and thyroid cancers were more frequent in females. Discrepancies between the Bavarian Cancer Registry and our data, such as higher rates of malignant melanoma (3400/63,771, 5.3% vs 1921/17,885, 10.7%) and lower representation of colorectal cancers (8100/63,771, 12.7% vs 1187/17,885, 6.6%) likely result from differences in the time periods analyzed (2019 vs 2021/2022) and the scope of data sources used. The Bavarian Cancer Registry reports approximately 3 times more cancer cases than the 6 university hospitals alone.

**Conclusions:**

The modular pipeline successfully transformed oncological RWD across 6 hospitals, and the federated approach preserved privacy while enabling comprehensive analysis. Future work will add support for recent oBDS versions, automate data quality checks, and integrate additional clinical data. Our findings highlight the potential of federated health data networks and lay the groundwork for future research that can leverage high-quality RWD, aiming to contribute valuable knowledge to the field of cancer research.

## Introduction

Real-world data (RWD), including information from various sources such as administrative claims data, electronic health records (EHRs), and cancer registries, offers a broad perspective on real-world patient populations, beyond the tightly regulated environment and specific conditions of randomized controlled trials [1-3]. RWD enables the generation of real-world evidence (RWE) concerning patient care by providing a comprehensive understanding of how interventions perform in real-life clinical settings and in diverse and unselected patient populations. This includes individuals often beyond the scope of randomized controlled trials, such as patients with frailty or comorbidities, or pregnant women, regardless of their social, cultural, or educational background [4-9].

In recent years, several RWD networks have emerged, designed to maximize the use of EHR data for research in medicine. While some of these networks cast a wide net, covering diverse clinical data sources, others have been more targeted, concentrating on data specific to certain diseases. The COVID-19 pandemic, for instance, led to the development of multiple dedicated data platforms, such as the National COVID Cohort Collaborative (N3C) [10], the 4CE consortium [11], and the CODEX platform [12]. Specialized clinical registries have been established worldwide to address specific health care needs [13]. In Germany, examples include the AKTIN Emergency Department Data Registry [14] and the Federal Clinical Cancer Registries [15], both of which focus on particular health care domains. Broader initiatives, like PCORnet [16], integrate data from EHRs, insurance claims, and patient-reported information to support research across various diseases. Other major networks, such as the Swiss Personalized Health Network (SPHN) [17] and the Observational Health Data Sciences and Informatics (OHDSI) initiative [18], work similarly to integrate and analyze large-scale health data.

In Germany, the Medical Informatics Initiative (MII) has established a large-scale data sharing network [19] based on electronic health record data from university hospitals, using the Health Level 7 (HL7) Fast Health care Interoperability Resources (FHIR) standard for data integration [20]. Hospitals harmonize heterogeneous clinical data in local data integration centers (DICs) nationwide, and a central portal has been established to access this data [21]. However, oncological data have not yet been integrated into the MII network. In Bavaria, the 6 university hospitals have united to form the Bavarian Cancer Research Center (BZKF) to provide comprehensive access to the latest methods of early detection, prevention, diagnosis, and treatment of cancer and build networked structures for cutting-edge research with a broad impact for all patients in Bavaria.

In this context, their oncology departments together with the 6 university hospitals` DIC have established a federated observational research network, building on the groundwork laid by the MII. Analyzing data from multiple hospitals enhances statistical validity by increasing the sample size, which enables rare event analysis in more diverse patient populations. However, the challenge of data protection in multisite scenarios underscores the need for implementing federated and privacy-preserving methods in data analysis [22-24].

This study aims to outline the design, implementation, and deployment of a modular data transformation pipeline that transforms oncological RWD into an HL7 FHIR format and then into a tabular format in preparation for a federated analysis (FA) across the 6 BZKF university hospitals.

## Methods

### Overview

In previous work, we detailed the necessary adaptations and extensions of existing MII components with the goal to enable federated feasibility queries on clinical oncology data [25], setting the groundwork for the BZKF Oncology Real World Data Platform. Our current goal is to extend the Oncology Real World Data Platform and implement a data transformation pipeline with an initial use case of performing an FA with a particular focus on data quality and comparability between the sites.

As a source of RWD, we use output from the 6 hospitals’ tumor documentation systems. Four of the hospitals use the same commercial system (ONKOSTAR) [26], whereas 2 hospitals apply a system called CREDOS, a tumor documentation system closely integrated into their EHR system, which was developed by one of the German Comprehensive Cancer Centers [27]. Because of the German law on national cancer registry data (Bundeskrebsregisterdatengesetz), both systems have to be able to export data in the oncological basic dataset (oBDS) format, a standardized dataset definition used nationwide for the collection of cancer data in cancer registries [28-30]. Since data pseudonymization is an important step in our pipeline, the respective pseudonymization tools already applied within the hospitals’ DIC (twice entici [31] and 4 times gPAS [32-34]) had to be generically integrated into the pipeline. Further, the pipeline end point was set as the DataSHIELD OPAL database, as we chose to use the privacy-preserving DataSHIELD framework [35] as our FA environment.

In designing our system architecture, because of the 6 sites’ heterogeneous software mix and our aim to keep our approach scalable for future deployments in additional hospitals with other systems, we established the following key objectives in accordance with related work in the field of FA in health care [22,23,36-39]:

- Modular adaptability: Create a flexible architecture to address diverse site requirements with regards to data extraction
- Multi-institutional FA: Data remains on site; only aggregated results are shared
- Security and privacy: Secure and nondisclosive analysis of pseudonymized patient data
- Interoperability: Enhance standard conformity by using HL7 FHIR, improving data management and stewardship
- Open source: Use open-source software for (cost) efficiency, longevity, community collaboration, and transparency.

To test the functionality and validity of our implementation, we defined a cohort to address specific medical research questions. We planned to include all patients who were diagnosed with cancer in 2022 and reported to the cancer registry as our data foundation for the following research questions:

- Q1: What is the distribution of tumor entities across the 6 university hospitals for cases diagnosed in 2022?
- Q2: What is the distribution of the administrative gender among the cases of tumor entities diagnosed in 2022?

To evaluate our pipeline, we compared the FA results with reports from the Bavarian Cancer Registry and with the original data from the local tumor documentation systems.

### Ethical Considerations

This retrospective study was approved by the relevant ethics committees and permission for data use was obtained from the use and access committees across all sites. All data was pseudonymized, and due to the FA method, it remained within its originating hospital and was never centrally pooled. In accordance with §27 of the Bavarian Hospital Act (Bayerisches Krankenhausgesetz), hospitals are permitted to use patient data for in-house research purposes, so no informed consent was required nor was any compensation provided.

- University Hospital Erlangen: application 23-1601-Br approved on September 21, 2023
- Klinikum rechts der Isar of Technical University of Munich: approval from University Hospital Erlangen is sufficient
- University Hospital Würzburg: approval from University Hospital Erlangen is sufficient
- University Hospital LMU Munich: application 23-0559 approved on November 14, 2023
- University Hospital of Augsburg: ethics committee of the University Hospital LMU Munich, application 23-0583 approved on November 28, 2023
- University Hospital Regensburg: application 23-3587-104 approved on December 5, 2023

## Results

### Project Workflow

**Figure 1:**
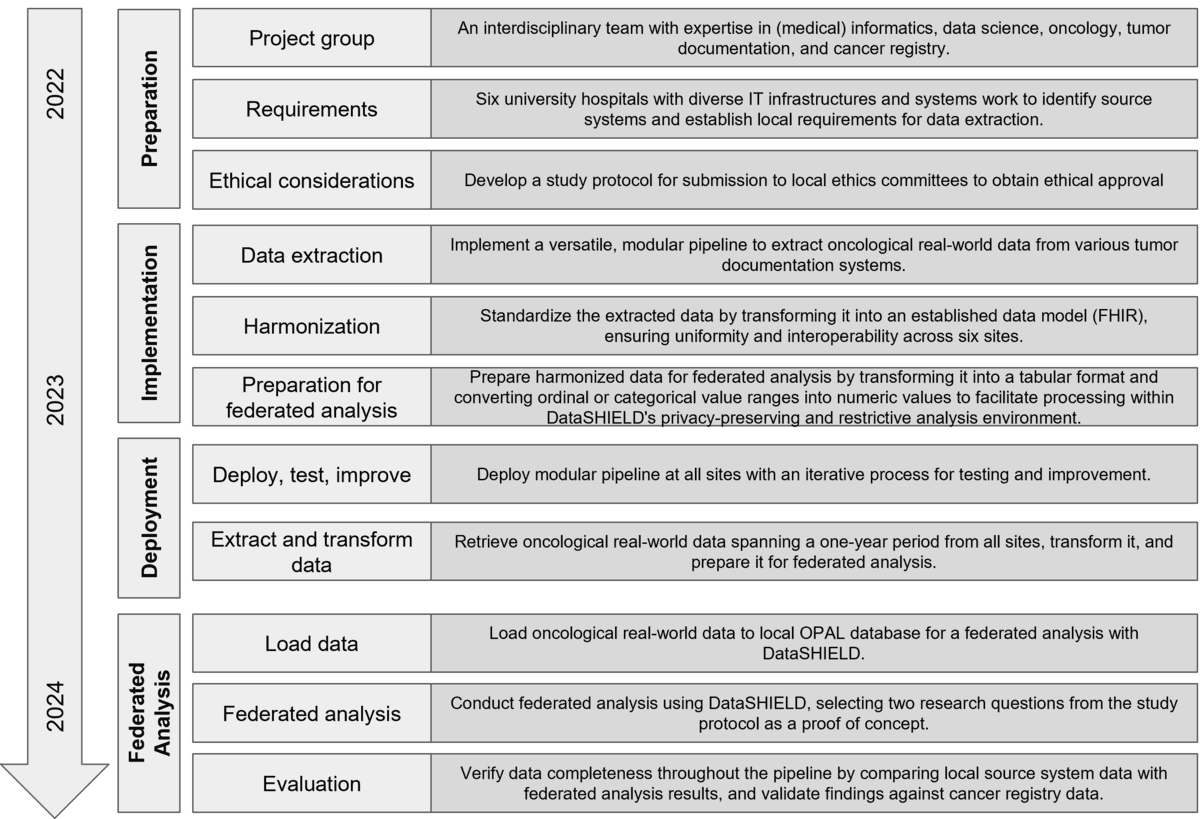
Project workflow diagram detailing each step of the process, providing a visual representation of the methodology and key activities involved.

Figure 1 presents a comprehensive workflow diagram of the project.

### Architecture

**Figure 2:**
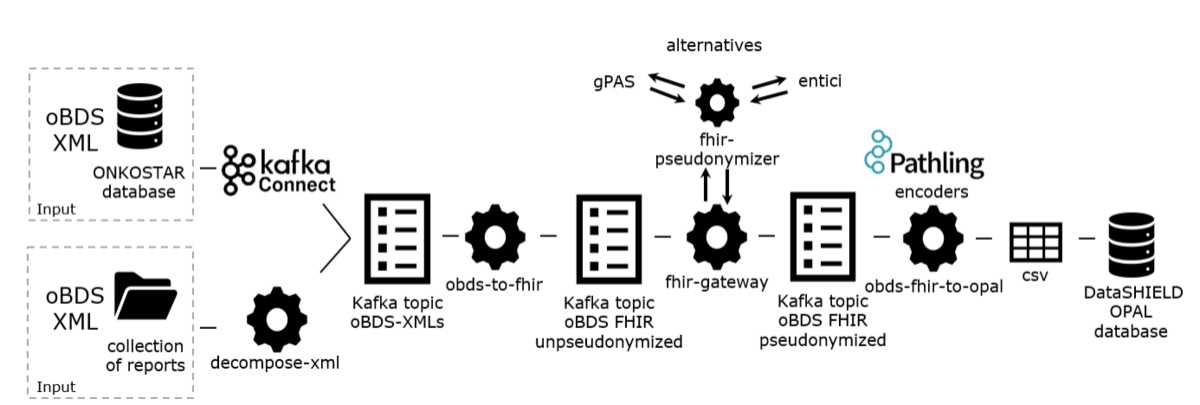
Architecture of the pipeline for transforming oncological basic dataset (oBDS) data into a final analysis format.

The complete pipeline architecture comprises 5 major modules and 4 transformation steps (Figure 2) and is described in more detail in the subsequent sections.

Figure 2 illustrates the key components, including 2 input interfaces, generic integration with pseudonymization services, and support for 2 output formats, enabling the conversion of oBDS data from XML to HL7 FHIR and to a tabular format suitable for FA.

#### Input Interfaces: ONKOSTAR Database Connector and Decompose-XML Folder Import

We incorporated 2 input interfaces: one that connects directly to the ONKOSTAR database, and another that functions through a folder import mechanism for locations without ONKOSTAR or access to its database. All tumor documentation systems offer an export of reported oBDS collections encoded in XML, the official format in which they are transmitted to state-mandated cancer registries. The oBDS collections differ structurally from the oBDS single reports stored in the ONKOSTAR database. Therefore, we provide a preprocessing service that reads in oBDS collections from a folder and decomposes them to match the format of single reports. As a second input interface, we provide an Apache Kafka Connect [40] connector to directly read in oBDS single reports from the ONKOSTAR database. In both import scenarios, an Apache Kafka producer [40] writes the (decomposed) single report as XML to a Kafka topic to be processed by the subsequent services.

#### Mapping Oncology RWD to FHIR: obds-to-fhir

We developed an extract-transform-load (ETL) process, a data integration method that involves extracting data from its original source, transforming it into a suitable format, and loading it into a target system. This process transforms oBDS XML-data to HL7 FHIR resources [41]. This component reads single oBDS XML-reports from an Apache Kafka topic, maps them to FHIR resources of the oncology FHIR model developed by Lambarki et al [42] and publishes the results to another Apache Kafka topic.

#### Pseudonymization: FHIR Gateway and FHIR Pseudonymizer

To deidentify the resources generated by the obds-to-fhir job, we deploy 2 services: the FHIR Gateway [43] and the FHIR Pseudonymizer [44]. The former reads resources from a given Kafka topic and sends them to the latter for pseudonymization based on configurable deidentification rules. For pseudonym generation, 4 sites use the pseudonymization service gPAS and 2 sites use the entici software, which we integrated with the FHIR Pseudonymizer. The FHIR Gateway publishes the resulting pseudonymized FHIR resources to a new output topic.

#### Transformation to Tabular Data: obds-fhir-to-opal

In the previous step, pseudonymized FHIR resources have been generated which can be used as the endpoint for feasibility queries as illustrated in our previous work [25]. The DataSHIELD FA framework however with its OPAL database requires a tabular data format [35]. Therefore we use the Pathling library FHIR encoders [45] in the obds-fhir-to-opal service to transform the nested FHIR resources into structured, tabular data. The library builds upon Apache Spark to convert FHIR bundles into Spark datasets. Following successful transformation of FHIR resources to dataframes, we use SQL and Spark functions for joining and grouping of relevant data elements tailored to the research queries. The result is a CSV file.

#### Upload to OPAL and Federated Analysis With DataSHIELD

**Figure 3:**
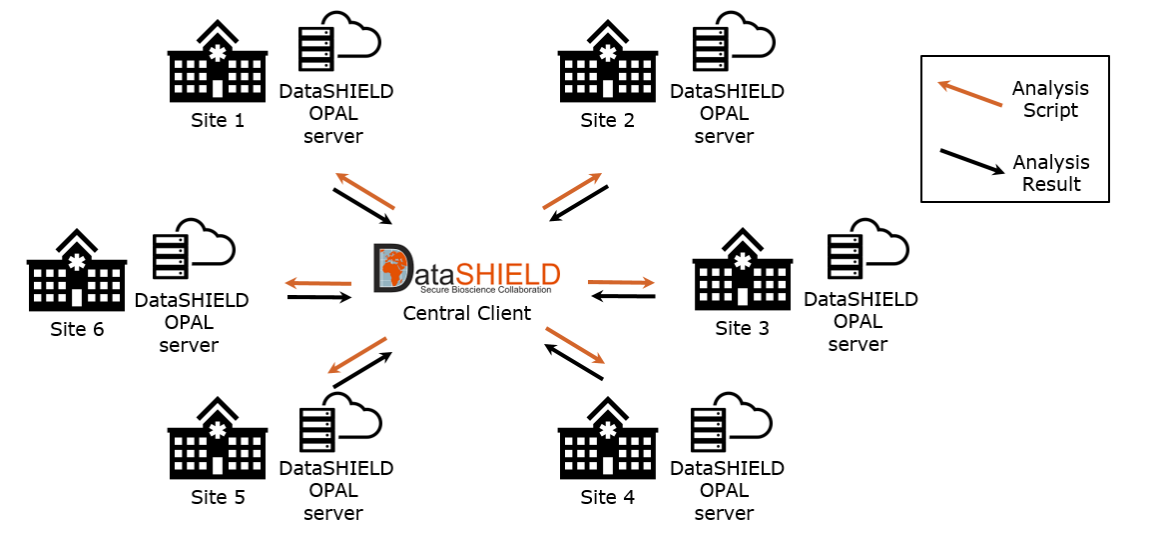
The federated analysis network illustrating the OPAL servers as the local analysis end points at each of the 6 Bavarian Cancer Research Center (BZKF) sites.

In the final step, we upload the CSV file resulting from the obds-fhir-to-opal service to the local OPAL servers. Figure 3 shows the FA network where the OPAL servers form the local analysis end points within each of the 6 BZKF sites. A central DataSHIELD client manages FA processes by distributing the analysis script across the network sites. These scripts are then locally executed, accessing the oBDS data stored in the local OPAL servers and returning aggregated results to the central DataSHIELD client, thus ensuring the confidentiality of private data by design.

#### Software Distribution to All Locations

We distribute the previously described software components from a public GitHub repository and the GitHub Container Registry [46]. Apart from a full setup, we provide multiple Docker Compose files which allow for a modular deployment of each individual component, enabling an easily adaptable setup at all sites and a generic integration with the different software systems already available at the sites (eg, ONKOSTAR, CREDOS, gPAS, entici). In addition, we supply Helm charts, which allow for the deployment and orchestration of all containerized applications in a Kubernetes cluster [47,48]. As several sites deploy the software on servers without internet connectivity, we provide an air-gapped installer that includes all container images compressed into an archive file for convenient download.

### Federated Analysis of Oncology Data

To address the two research questions, we used the data elements *International Classification of Diseases, 10th Revision* (*ICD-10*) diagnosis code, date of diagnosis, and gender. We aggregated all diagnoses from 2022 for sites 1-5. For site 6, only data from 2021 was available and therefore used.

The total volume of cancer data analyzed for the 1-year time span across all 6 BZKF sites comprised 17,885 patients, including 7969 women, 9913 men, and 3 individuals of other or unknown genders. The 10 most frequent cancer diagnoses included prostate cancer (n=2476, 13.8%); breast cancer (n=2006, 11.2%); malignant melanoma of the skin (n=1921, 10.7%); cancer of the trachea, bronchus, and lungs (n=1415, 7.9%); cancer of the lip, oral cavity, and pharynx (n=1329, 7.4%); and cancer of the colon and rectum (n=1187, 6.6%). Non-Hodgkin lymphoma (n=801), cervical cancer (n=719), pancreatic cancer (n=644), and thyroid cancer each accounted for 4%.

In the latest report for the year 2019, the Bavarian Cancer Registry reported a total of 63,771 cancer diagnoses with the following 10 most frequent entities: breast cancer (n=11,260, 17.7%); prostate cancer (n=8590, 13.5%); cancer of the colon and rectum (n=8100, 12.7%); cancer of the trachea, bronchus, and lungs (n=5821, 9.1%); malignant melanoma of the skin (n=3400, 5.3%); bladder cancer (n=3253, 5.1%); and cervical cancer (n=3030, 4.8%). Pancreas cancer (n=2214), non-Hodgkin lymphoma (n=2133), and stomach cancer (n=2023) each represented 3% of the cases.

**Figure 4:**
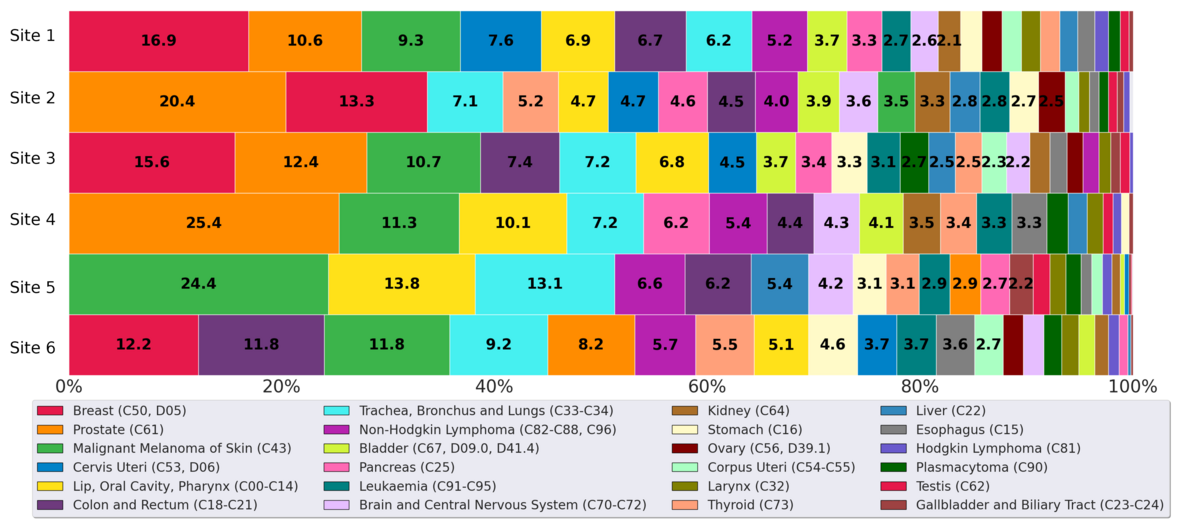
Distribution of tumor entities at each hospital for cases diagnosed in 2022 (site 1-5) and 2021 (site 6). Results of the federated analysis across 6 locations in relative numbers per site (research question Q1).

Figure 4 illustrates the distribution of cancer incidences among various cancer types diagnosed in 2022 (site 1-5) and 2021 (site 6) within the BZKF (research question Q1). Both breast cancer (C50, D05) and prostate cancer (C61) rank among the top 2 in 5 sites, with breast cancer being the most prevalent in 3 of these sites and prostate cancer being the most prevalent in 2. Malignant melanoma of the skin (C43) also shows a significant representation, particularly in site 5 with 24.4% (500/2045).

Site 4 reported no instances of breast cancer or uterine cancers, as its gynecology department does not use ONKOSTAR nor CREDOS and therefore has not yet been integrated into our pipeline. Furthermore, site 5 exhibited notably fewer cases of gynecological cancers (breast, cervix, and uterus), as the university professorships for gynecology and obstetrics are based at affiliated hospitals separate from the university hospital, and thus, this data was not fully accessible for our analysis. This site reported the lowest relative number of prostate cancer cases, likely because the Department of Urology is also based at a partner hospital. As a result, the urological cancer data from site 5 is probably incomplete in our dataset. Site 6 showed a lower prevalence of prostate cancer and a relatively higher prevalence of colon and rectal cancers compared with the other sites. This site did not report any cases of testicular cancer.

**Figure 5:**
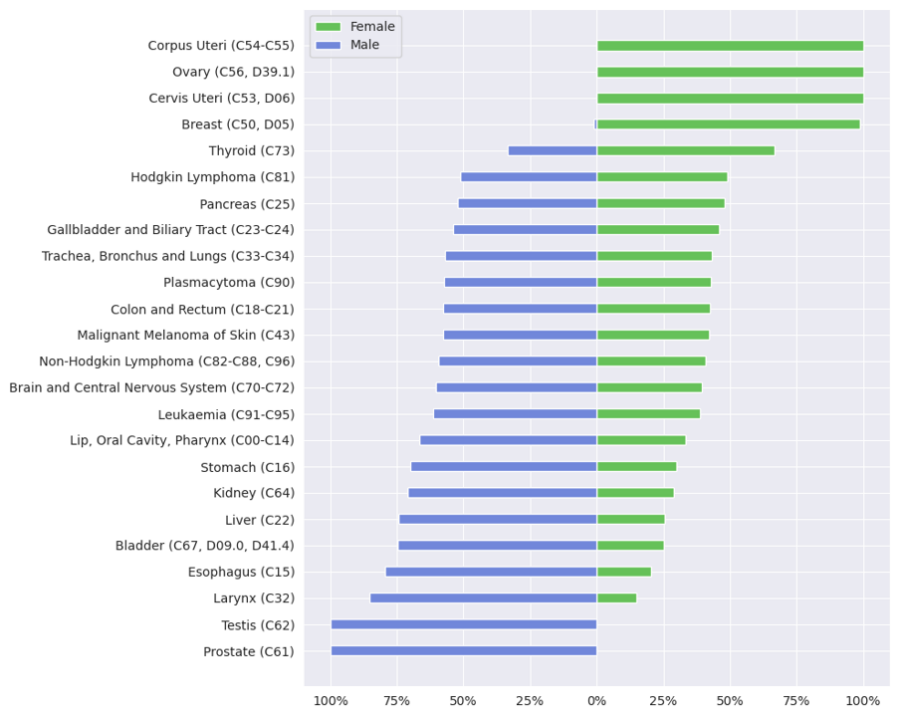
Distribution of administrative gender among the tumor entities for cases diagnosed in 2022 (site 1-5) and 2021 (site 6). Mean results of the federated analysis across 6 locations in relative numbers (research question Q2). Other genders are omitted from the visualization due to the presence of only 3 cases.

Figure 5 presents an overview of how different cancer diagnoses are distributed among female and male patients in 2022 (site 1-5) and 2021 (site 6). It depicts the aggregated frequencies of cancer diagnoses for each entity group across all 6 locations and highlights relative distribution for female and male patients (pertaining to research question Q2). Apart from cancer affecting sex-specific organs, such as cancers of the prostate and uterus, there are notable differences in the frequency of other cancer diagnoses between sexes.

Cancer types such as larynx, esophagus, bladder, liver, kidney, or stomach see higher frequency rates in males compared with females, a trend also observed with cancers of the lip and oral cavity as well as leukemia, which are predominantly diagnosed in males. In contrast, breast and thyroid cancer frequency is higher in females. These findings are consistent with the reviewed literature, which explored sex differences in cancer incidence, including data from the latest Robert Koch Institute report, which details cancer incidence across Germany [49]. It is important to note, however, that while the reports present incidence rates, our data depict frequency distributions; thus, the figures are not directly comparable but instead offer a general indication of trends. This report mentions a higher incidence of breast cancer and a slightly higher incidence for thyroid cancer for women, and generally a higher incidence for many other cancers, including larynx, esophagus, and bladder for men. This gender-based difference aligns with studies in the United States; Kim et al [50] found that prostate, lung, and colorectal cancers are among the most frequent cancer diagnoses in males, while breast, lung, and colorectal cancers predominate cancer diagnoses in females. The trend shown for lung and colorectal cancers in female patients is not reflected in our data from the 6 Bavarian university hospitals. They noted a significantly higher incidence of thyroid cancer in females and highlighted that colorectal, stomach, and liver cancers, as well as bladder cancer and leukemia, occur more often in males. Further supporting these findings, Jackson et al [51] demonstrated that male cancer incidence is higher across many cancer types, with significant male-to-female hazard ratios for cancers like bladder, gastric cardia, larynx, and esophageal adenocarcinoma, with ratios ranging up to 10.8 times higher in males. This study also identified lifestyle and environmental risk factors, such as smoking and alcohol, contributing to the observed sex disparities for cancers of the liver, biliary tract, bladder, skin, colon, rectum, and lung. Harvey and Harvey [52] examined global data from the Global Cancer Observatory and reported that, beyond cancers that occur exclusively in one sex due to anatomical differences, males show higher age-standardized incidence and mortality for almost all cancer types. They suggest that hormonal and genetic factors, such as estrogen’s role in colon cancer, as well as nonbiological risk factors, like smoking and alcohol, contribute significantly to these disparities in cancer incidence and mortality rates across genders.

**Figure 6:**
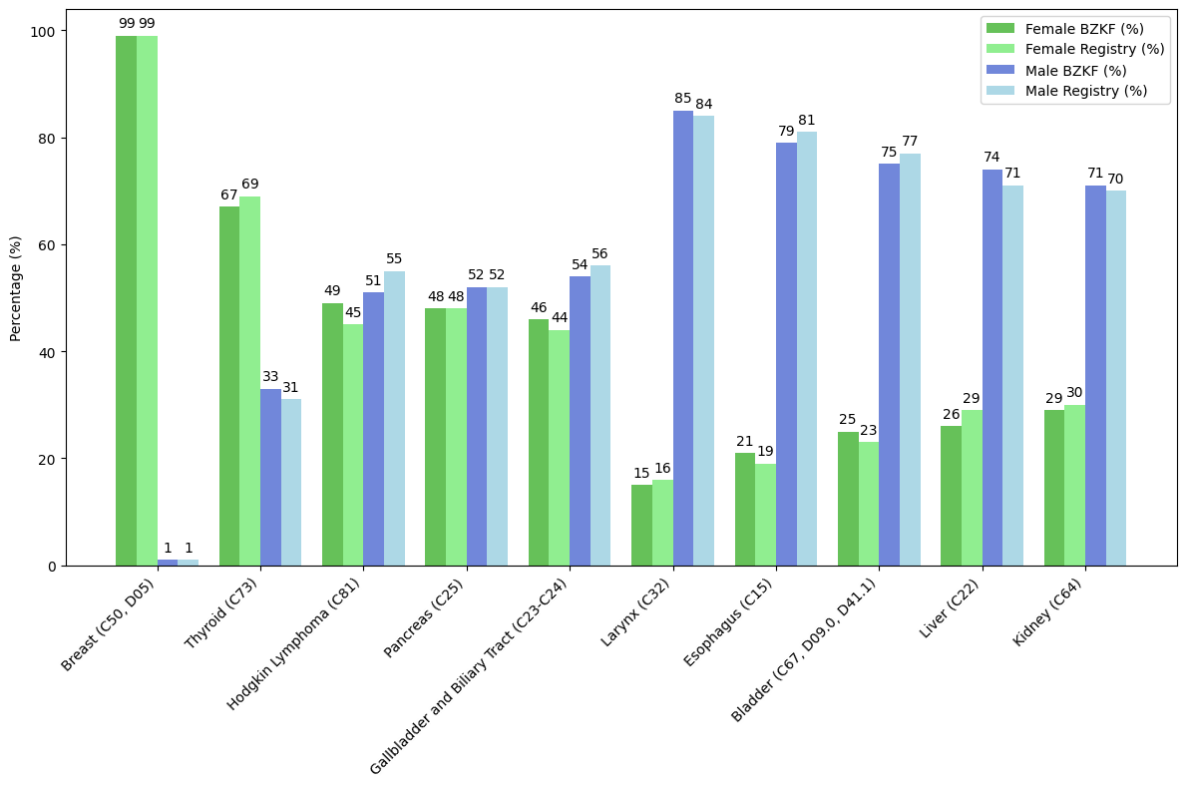
Distribution of the top 5 most frequently diagnosed conditions among female and male patients, excluding sex-specific organs (uterus, ovary, prostate, and testis).

We compared our results with the 2019 Bavarian Cancer Registry report and identified the distribution of the 5 most frequently diagnosed conditions among female and male patients, focusing on the gender distribution of each specific condition and excluding sex-specific organs (Figure 6). The figure highlights the gender-specific distribution of each condition, comparing data from the Bavarian Cancer Research Center (BZKF, 2021/2022) with the Bavarian Cancer Registry report (2019).

### Comparison of Federated Analysis Results With Tumor Documentation Systems

To evaluate validity, we compared the total number of diagnoses in the original data from the local tumor documentation systems to the total number of diagnoses after being processed by the presented pipeline and aggregated through the FA framework Data SHIELD. The following describes the calculation of the entity-wise deviation (mean absolute percentage error):

*ŷ*: predicted value for entity *i* (federated analysis result)

*y*: gold standard for entity *i* (evaluation with tumor documentation system)

For each entity i, calculate entity-wise deviation and return mean value over all 24 entity-wise deviations (mean absolute percentage error):



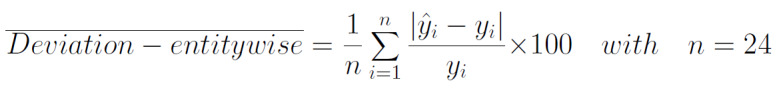



The evaluation was conducted by tumor documentation specialists querying the tumor documentation systems or using a custom built tool that automates the majority of the process [53].

For sites 1, 2, 3, and 6, the mean of entity-wise deviations remained under 2% (1.6%, 1.9%, 1.4%, and 1.22%, respectively), contrasting with sites 4 and 5, which exhibited a mean of entity-wise deviation of 3.7% and 11.6%, respectively.

## Discussion

### Principal Findings

Previous research has highlighted the importance of using RWD for generating RWE on patient care in diverse, unselected populations [1-9,21]. Geldof et al [54] have argued for the development of federated RWD infrastructures on a common data model, capable of bringing the centrally conducted big data analysis to the decentrally stored biomedical data. Following this paradigm, the BZKF multi-institutional research network offers the foundation to leverage insights into oncology RWD from 6 sites. However, our process of creating a harmonized foundation of care-related RWD from tumor documentation systems across the BZKF university hospitals with heterogeneous IT infrastructures also illustrated challenges arising in such real-world environments.

We have outlined the successful development and deployment of a modular pipeline for extracting, harmonizing, and transforming oBDS data across the 6 BZKF university hospitals. Unlike traditional statewide cancer registries, which centralize data collection and analysis, our approach uses FA, keeping data decentralized and preserving privacy by design.

We demonstrated the functionality of our pipeline through an FA using the DataSHIELD framework to address two research questions. Our analysis shows breast cancer (C50, D05) as the most common at 3 sites and prostate cancer (C61) among the top 2 at 4 sites. In addition, cancers of the larynx, esophagus, bladder, and liver are more frequent in males, while breast and thyroid cancers are more common in females (excluding sex-specific cancers).

Our findings generally align with expected incidence rates or can be attributed to local specializations in treatment and data availability [49-52,55]. However, our data shows a slightly higher frequency of malignant melanoma of the skin (11%) compared with the Bavarian Cancer Registry (5%) [55]. Conversely, colorectal cancers are underrepresented in our data (7%) compared with the registry (13%) [55]. These discrepancies might be partially attributed to the broader data sources used by the Bavarian Cancer Registry, which include additional clinics, outpatient facilities, and other reporting institutions, ultimately reporting approximately 3 times more cancer diagnoses than the BZKF, as well as the different time periods analyzed (2019 for the registry vs 2021/2022 for our study).

In light of the comparison of our reported figures and the data recorded in the tumor documentation systems, several factors may account for the discrepancies observed. One significant issue is the occurrence of retrospectively documented cases. These arise in cases with a large time delay between diagnosis and documentation or if cases initially diagnosed externally are later incorporated. The latter situation arises when patients, who were diagnosed elsewhere in the relevant time period but are now receiving treatment at one of the 6 facilities, have their therapy documented now with a diagnosis date in the past within the relevant time period.

Another contributing factor is the timing mismatch between data extraction and the data quality evaluation. Data extraction was performed in January, while the evaluation occurred in May. This delay may have led to an increase in cases recorded in the tumor documentation systems due to the reasons outlined above. In addition, re-extraction of data was not feasible for site 5, which showed the highest entity-wise deviation of 11.6%. This site had transitioned to oBDS version 2.2.3 in February, while our pipeline only supports up to oBDS version 2.2.2, preventing us from processing the updated data from this site.

Moreover, we found a discrepancy between the data elements defined in the oBDS standard and those available in the oBDS XML files reported to the cancer registry. We had intended to investigate the Union for International Cancer Control (UICC) stage of cancer diagnoses, but this data was largely missing in the oBDS reports from most sites. Since we currently only process oBDS data from XML reports sent to the cancer registry, our dataset could be enhanced by extracting additional data elements from other database tables within the tumor documentation systems, leveraging even more of the documented data. However, this is not feasible with the current decompose-XML folder import interface, which is limited to reading oBDS XML reports. If expanding the dataset in this manner is a future goal, we would need to establish direct access to the tumor documentation databases at all locations to retrieve additional data beyond the oBDS XML reports. The transition of 2 locations from CREDOS to ONKOSTAR would help streamline this process by eliminating the use of two different systems.

### Lessons Learned

Health care research IT infrastructure requires tailored solutions and adherence to established processes and security standards. Our work highlighted several challenges stemming from heterogeneous IT systems across sites. Certain locations require air-gapped installations, isolated from unsecured networks to protect sensitive data, complicating development, deployment, and maintenance. In addition, the DataSHIELD framework imposes strict restrictions on analytics to ensure data privacy. To address these issues, we iteratively adapted the obds-fhir-to-opal module, implementing various groupings and mappings directly into the dataset, which was crucial for effective analysis within the framework’s constraints. Significant challenges such as data incompleteness, the use of various documentation systems, and the heterogeneity of documentation practices across different hospitals or subclinics per site persist. Similar to Maier et al [56], we found that it is an essential requirement to have precise information about the conditions under which documentation was conducted and in what time frame after the original event documentation is pursued.

We also learned that data from some sites should not be integrated into future analysis of dedicated cancer entities (eg, breast and prostate cancer) since their provided dataset is not representative because of local organizational structures or the documentation in a particular clinic still being pursued with a tumor documentation system not yet integrated into our pipeline. Thus, our insights add further perspectives to the barriers to RWD analysis mentioned by Saesen et al [57] (methodological and operational challenges), illustrating that the knowledge about the documentation practice, context, and potential incompleteness of RWD integrated into a RWD network is essential to avoid misinterpretation of analysis results.

### Future Work

We plan to support future oBDS versions in our ETL job. To achieve this, the ETL job was made open source, and we are building a development community across interested parties to improve and extend the ETL job and the pipeline. To date, data quality and completeness checks have predominantly depended on human intervention. Ru et al [58] highlighted the absence of interoperable data quality standards and observed significant variability in the quality of two RWD networks following data quality assessment. The inclusion of data from 6 sites introduces even more variability and further underscores the significance of addressing data quality and completeness. Alongside addressing future queries, we will develop a unified evaluation strategy that incorporates automated data quality and plausibility checks into the pipeline, aligning with the standards of the State Cancer Registry [59] for completeness, validity and plausibility such as ensuring date variables follow a logical sequence (eg, birth date ≤ diagnosis date) or verifying valid combinations of histology, tumor localization, and TNM staging. To achieve this, we plan on using great expectations [60] along with previously developed DQ solutions [61] to implement the checks and execute them continuously within the pipeline.

Berger et al [9] emphasize the critical need to integrate various often siloed RWD sources to produce high-quality RWE in oncology. Addressing this gap involves incorporating RWD such as laboratory findings, pathology reports, radiology reports and molecular genetic data from molecular tumor boards. Converting data to the FHIR data model enhances interoperability across systems and sites and facilitates the integration of these in the past-siloed data sources at the DIC. Thus, the next steps for the BZKF sites will involve integrating the various sources of oncology data with the oBDS datasets leveraged within our project and the MII core dataset data [62], already available within the DIC. Inspired by the findings of Swinckels et al [63], who showed in their scoping review of 20 studies that machine learning and deep learning applied to longitudinal EHR data can greatly enhance early disease detection and prevention across various conditions, we plan to integrate various data sources and analyze longitudinal data from the past decade. This approach will enable us to conduct more comprehensive analyses and develop machine learning models for detecting or predicting oncological diseases. Expanding the number of hospitals involved is also essential to increase sample size and diversify patient populations. Therefore, we will contribute our open source pipeline as well as our experiences and insights into ongoing work within oncology-related Germany-wide projects, such as the expansion of the national portal for medical research data [21] and the MII project Personalized Medicine for Oncology (PM4Onco) [64].

Building on our findings, we summarize our future efforts as follows: supporting all oBDS versions, automating data quality checks, integrating additional data sources, analyzing longitudinal data, and scaling and collaboration.

### Conclusion

Our modular approach demonstrates the feasibility of converting oncological RWD into HL7 FHIR and tabular data and querying it in a federated way across 6 sites. These findings motivate us to build on this work and integrate the full set of oBDS data from the 6 university hospitals to leverage the value of more than 200,000 oncological cases from the last decade in the future, growing by about 20,000 new cases annually. The dataset can be leveraged for cohort searches, hypothesis generation, study planning, and the development of new AI models. In their 2021 systematic review of research applications of FA, Hunger et al [65] emphasized that additional efforts are necessary to promote awareness about the significant potential of FA in leveraging readily available RWE to address key research questions in cancer. Our study contributes to achieving this goal, and we will continue to explore the benefits of FA for RWD in our future research. Through a focus on iterative processes aimed at integrating further clinical data and improving data quality, we aim to generate valuable RWE from previously untapped sources of care-related information, ultimately aiming to make significant contributions to cancer research.

## Abbreviations

BZKF: Bavarian Cancer Research Center
CREDOS: Cancer Retrieval Evaluation and Documentation System
DIC: Data Integration Center
EHR: electronic health record
ETL: extract-transform-load
FA: federated analysis
FHIR: Fast Healthcare Interoperability Resources
HL7: Health Level 7
ICD-10: International Classification of Diseases, 10th Revision
MII: Medical Informatics Initiative
oBDS: Oncological Basic Data Set
OHDSI: Observational Health Data Sciences and Informatics
PM4Onco: Personalized Medicine for Oncology
RWD: real-world data
RWE: real-world evidence
SPHN: Swiss Personalized Health Network
UICC: Union for International Cancer Control

## Data Availability

The original and processed data are not publicly available due to privacy restrictions. The software can be retrieved from the GitHub repository [46].
